# Locked-Window EQ-5D-5L (Index and VAS) Benchmarking in Sarcoma Care: Rule-Based Traffic-Light Classification Across Two Institutions

**DOI:** 10.3390/diseases14050159

**Published:** 2026-04-30

**Authors:** Isabel Gloor, Beatrice Meier, Jehona Rexhai, Philip Heesen, Georg Schelling, Bettina Vogel, Gabriela Studer, Bruno Fuchs, on behalf of the Swiss Sarcoma Network

**Affiliations:** 1 Department of Orthopaedics and Trauma, LUKS Sarcoma-IPU, University Teaching Hospital LUKS, Spitalstrasse, 6000 Lucerne, Switzerland; 2 Department of Orthopaedics and Trauma, Kantonsspital Winterthur, KSW Sarcoma Center, Brauerstrasse 15, 8401 Winterthur, Switzerland; 3 Faculty of Medicine, University of Zurich, Raemistrasse 71, 8006 Zurich, Switzerland; 4 Swiss Sarcoma Network SSN, LUKS Sarcoma-IPU, University Teaching Hospital LUKS, Spitalstrasse, 6000 Luzern, Switzerland; 5 Department of Radiation Oncology, LUKS Sarcoma-IPU, University Teaching Hospital LUKS, Spitalstrasse, 6000 Lucerne, Switzerland; 6 Faculty of Health Sciences & Medicine, University of Lucerne, Frohburgstrasse 3, 6002 Luzern, Switzerland

**Keywords:** sarcoma, patient-reported outcomes, EQ5D-5L, quality of life, benchmarking, value-based healthcare, survivorship, learning health system, quality improvement, real-world-time data

## Abstract

Background: Value-based sarcoma care requires outcome measures that reflect the patient perspective; however, many sarcoma episodes begin with near-normal function and undergo necessary morbidity for oncologic control, making simple “improvement” an unreliable proxy of value. In routine care, patient-reported outcome data are often irregular and incomplete, limiting benchmarking and learning across institutions. We therefore developed a rule-based EQ-5D-5L (index and VAS) traffic-light framework and evaluated its feasibility and benchmarking signal in two institutions. Methods: We performed a retrospective, two-institution cohort analysis of 729 malignant and intermediate episodes, defined using a prespecified histology behavior mapping. PROM evaluation was anchored to a hierarchical T0 (index surgery date; if unavailable, radiotherapy start date; if unavailable, systemic therapy start date where a valid and interpretable start date was available). EQ-5D-5L index and EQ-VAS were assigned to prespecified locked windows: baseline (−90 to +14 days preferred; +15 to +90 days fallback), 12 months (180–365 days; target 270), and 24 months (660–820 days; target 730). A rule-based traffic-light classification was applied at 12 and 24 months (RED if index < 0.75 or VAS < 50; GREEN if index ≥ 0.85 and VAS ≥ 70; otherwise YELLOW). PROM evaluability was defined as the availability of at least one valid EQ-5D-5L index and/or EQ-VAS value within each window. Results: PROM evaluability in locked windows was feasible but incomplete. Baseline PROMs were available for 107/729 episodes (14.7%), 12-month PROMs for 119/729 (16.3%), and 24-month PROMs for 84/729 (11.5%). At 12 months, evaluable episodes included 75 from Institution A and 44 from Institution B; at 24 months, 56 and 28, respectively. Traffic-light outputs showed heterogeneity at both timepoints and clearer cross-institution difference at 24 months than at 12 months. At 12 months, the distribution was predominantly GREEN in both institutions (Institution A: 73.3% GREEN, 9.3% YELLOW, 17.3% RED; Institution B: 65.9% GREEN, 18.2% YELLOW, 15.9% RED; *p* = 0.373). At 24 months, Institution A maintained a high GREEN proportion with a low RED fraction (76.8% GREEN, 17.9% YELLOW, 5.4% RED), whereas Institution B showed a lower GREEN proportion and higher YELLOW/RED fractions (50.0% GREEN, 25.0% YELLOW, 25.0% RED; *p* = 0.014). Absolute EQ-5D-5L medians remained high overall, but the follow-up distributions showed a broader lower tail in Institution B. Conclusions: A prespecified EQ-5D-5L (index and VAS) traffic-light framework anchored by hierarchical T0 and evaluated in locked windows yields interpretable patient-perspective benchmarking signals in real-world sarcoma care. The approach was operationally feasible within the evaluable subset and appeared more discriminative at 24 months than at 12 months, while incomplete PROM capture remains a major implementation limitation for representative and reliable network-scale benchmarking and learning.

## 1. Introduction

Sarcoma care poses a distinct challenge for value-based oncology because clinical benefit and patient-perceived benefit are often not aligned [1]. In many oncologic pathways, treatment is expected to reduce symptoms or prolong survival, albeit at the cost of toxicity. In sarcoma, however, definitive local therapy—most commonly surgery and frequently combined with radiotherapy and/or systemic therapy—is often necessary for disease control but may impose substantial, and sometimes irreversible, functional morbidity [2,3,4]. At the same time, many patients enter treatment with near-normal baseline function, such that “improvement” is not the expected direction of change. Outcome evaluation in sarcoma therefore requires a framework that can distinguish acceptable survivorship from patient-perceived unacceptable health states and that remains interpretable across heterogeneous treatment sequences and institutional pathways [5,6,7].

Patient-reported outcome measures (PROMs) are central to evaluating patient-perspective value, but their application in sarcoma is limited by inconsistent timing, incomplete capture, and substantial heterogeneity in follow-up structure [2,4]. Generic instruments such as EQ-5D-5L provide a pragmatic basis for network-wide benchmarking because they can be collected across diagnoses and treatment modalities and allow broad comparability [8,9,10,11,12]. However, real-world PROM data are typically sparse and irregular, and short-term postoperative or post-treatment fluctuations may obscure longer-term survivorship patterns [13,14,15]. Without explicit anchoring and prespecified evaluation windows [16,17], PROM comparisons across institutions risk becoming non-comparable moving targets, thereby limiting their usefulness for governance, benchmarking, and iterative quality improvement within learning systems [18,19,20,21].

Most sarcoma PROM studies report absolute health-related quality-of-life values at selected follow-up visits and, variably, change-from-baseline metrics [15,22]. While informative descriptively, such approaches are often difficult to operationalize for cross-institution benchmarking because denominators shift across timepoints, follow-up timing is heterogeneous, and missingness is rarely made explicit in a governance-ready way [23,24,25]. A PROM framework intended for value-based care requires four elements: anchoring to a clinically meaningful index event [16,26], prespecified evaluation windows [17,27], transparent evaluability denominators [23,28,29,30], and an interpretable output that can support routine decision-making [31,32]. In this context, the challenge is not merely to measure PROMs, but to render them operationally steerable [33].

To address these limitations, we developed and evaluated a prespecified, rule-based VOS PROM module using EQ-5D-5L index and EQ-VAS, anchored to a hierarchical start-of-definitive-treatment time zero (T0) and evaluated in locked windows at baseline, 12 months, and 24 months [8,9,10,21,24]. The module translates PROM status into an interpretable traffic-light output (green/yellow/red) that prioritizes unacceptable health states while accounting for meaningful change relative to baseline and for ceiling effects that are common in sarcoma pathways [34,35]. This approach was designed explicitly for feasibility and benchmarking signal detection rather than for causal attribution of treatment effects, while supporting transparent reporting of evaluability, denominators, and longitudinal stability between 12 and 24 months [36,37,38].

In this multicenter feasibility study, we applied the module to malignant and intermediate sarcoma episodes from two institutions and assessed (i) PROM evaluability under locked-window definitions, (ii) distributions of EQ-5D-5L index and EQ-VAS at baseline, 12 months, and 24 months, and (iii) the resulting traffic-light benchmarking signal across institutions, including 24-month stability patterns [39,40,41,42]. By operationalizing a patient-perspective value signal in real-world sarcoma care, this work provides a pragmatic methodological template for PROM-based governance and establishes a foundation for subsequent risk-adjusted benchmarking and pathway refinement, including incorporation of sarcoma-domain variables such as bone versus soft tissue disease and reconstruction exposure once cross-institutional harmonization is complete [43,44,45].

## 2. Materials and Methods

### 2.1. Study Design and Setting

This secondary analysis of prospectively—from October 2021 to April 2025—collected real-world data from two institutions assessed the feasibility and benchmarking signal of a rule-based patient-reported outcome (PRO) module embedded in a Value Operating System (VOS) for sarcoma care. The methodological objective was to translate routinely collected EQ-5D-5L measurements into an interpretable patient-perspective traffic-light output at clinically meaningful timepoints and to quantify cross-institution differences under a harmonized, prespecified evaluation framework. Data were provided as anonymized episode-level records from two institutions (Institution A and Institution B). Analyses were performed at the episode level, and results are reported separately for each institution and for the combined cohort.

### 2.2. Cohort Definition and Eligibility

Episodes were screened for inclusion based on two criteria: (i) histological diagnosis behavior category and (ii) availability of a valid time anchor (T0) enabling locked-window PROM evaluation.

#### 2.2.1. Histology Behavior Mapping

Histological diagnosis strings were mapped to a three-tier tumor-behavior framework (Benign, Intermediate, Malignant) using a prespecified rule set derived from recognized entity-level behavior. The mapping, including confidence rating and rationale per label, is provided in Table S1. The primary analytic cohort included episodes classified as Malignant or Intermediate, reflecting the intended clinical scope of sarcoma services, including locally aggressive or borderline entities that may require extensive local therapy and can generate substantial morbidity. Episodes classified as Benign were excluded.

Diagnosis strings that could not be assigned unambiguously to a behavior tier based on the available recorded label were flagged as Unclear and excluded from the primary analysis to avoid misclassification. The complete list of Unclear labels is included in Table S1 to enable transparent adjudication should definitive behavior coding (e.g., ICD-O behavior code or an explicit pathology statement) become available.

#### 2.2.2. Time-Anchor Eligibility

Only episodes with a valid hierarchical time anchor (T0; defined below) were eligible for time-window-based PROM evaluation. Episodes without a valid T0 were excluded from the locked-window analyses.

### 2.3. Patient-Reported Outcomes

Patient-reported health status was assessed using the EQ-5D-5L index utility score and the EQ visual analogue scale (EQ-VAS). PROMs were available at irregular timepoints in routine care, and episodes could contribute a single observation or repeated measurements over time. No imputation was applied for missing PROM data. Instead, PROM availability (“evaluability”) was quantified explicitly as part of the feasibility assessment.

### 2.4. Time Anchoring (T0) and Prespecified Evaluation Windows

#### 2.4.1. Hierarchical Definition of T0

For each episode, the time zero (T0) was defined hierarchically as the start of definitive treatment to enable consistent anchoring across heterogeneous treatment sequences. The recorded index surgery date was used when available. If the index surgery date was missing, the recorded radiotherapy start date was used. If both were missing, a recorded systemic anticancer therapy start date was used where a valid and interpretable treatment start date was available. Episodes lacking all three anchors were not eligible for time-window-based PROM evaluation. These anchors were selected as pragmatic reference points for standardizing PROM evaluation across heterogeneous real-world treatment pathways, not because surgery, radiotherapy, and systemic treatment starts were assumed to represent biologically or functionally equivalent states.

#### 2.4.2. Baseline Selection (Two-Step Rule)

Because true pre-treatment PROMs are not consistently available in routine care, baseline was defined pragmatically using a two-step selection rule that prioritized pre-treatment or peri-treatment measurement. First, the PROM assessment closest to T0 within −90 to +14 days was selected, with preference given to pre-treatment measurements where present. If no PROM was available in this preferred window, the closest PROM within the fallback window of +15 to +90 days after T0 was selected and flagged as an early-post baseline.

#### 2.4.3. Locked 12-Month and 24-Month Evaluation Windows

To ensure interpretability and reduce sensitivity to short-term postoperative fluctuation, PROM outcomes were evaluated at prespecified locked windows anchored to T0. The 12-month evaluation (“12 m”) was defined as the PROM measurement closest to 270 days within 180–365 days after T0. The 24-month evaluation (“24 m”) was defined as the PROM measurement closest to 730 days within 660–820 days after T0.

#### 2.4.4. PROM Evaluability

At each timepoint (baseline, 12 m, and 24 m), PROM evaluability was defined as the presence of at least one valid observation containing either an EQ-5D-5L index value and/or an EQ-VAS score within the respective time window. Evaluable *n* was reported per institution and timepoint.

### 2.5. VOS PROM Module: Rule-Based Patient-Perspective Classification

#### 2.5.1. Absolute Health-State Thresholds

At the locked follow-up timepoint (12 m or 24 m), absolute health state was classified using prespecified, operational thresholds informed by population norms and clinical reasoning [46,47]:

- EQ-5D-5L index: RED < 0.75; GREEN ≥ 0.85.
- EQ-VAS: RED < 50; GREEN ≥ 70.

Absolute state assignment followed conservative prioritization. Episodes were classified as absolute RED if either metric was below its RED threshold and as absolute GREEN only if both metrics met GREEN thresholds. All remaining patterns were classified as absolute YELLOW.

#### 2.5.2. Meaningful Change Relative to Baseline

Meaningful change relative to baseline was evaluated using prespecified minimal important difference (MID) thresholds [36,37,38]:

- ΔEQ-5D-5L index: ±0.03.
- ΔEQ-VAS: ±5.

Clinically meaningful improvement was defined as Δ ≥ +MID in either metric, and clinically meaningful deterioration as Δ ≤ −MID in either metric. All other patterns were classified as stable.

#### 2.5.3. Ceiling-Effect Handling

To address ceiling effects in episodes with high baseline function, analyses were stratified by baseline level using prespecified thresholds: EQ-5D-5L index ≥ 0.90 and/or EQ-VAS ≥ 80 to define a high baseline state. This stratification was used to distinguish maintained acceptability from clinically meaningful loss in episodes starting near the top of the scale.

#### 2.5.4. Final PROM Traffic-Light Assignment

The final PROM traffic light at 12 m and 24 m combined absolute state and meaningful change with explicit prioritization rules. Absolute unacceptability (RED) dominated: episodes meeting absolute RED criteria were assigned final RED irrespective of change. Episodes meeting absolute GREEN criteria were assigned final GREEN, except where clinically meaningful deterioration from a high baseline indicated potential value ambiguity; such episodes were conservatively downgraded to YELLOW. All remaining episodes were classified as YELLOW, unless clinically meaningful deterioration suggested transition toward unacceptable health status, in which case RED was assigned.

#### 2.5.5. Stability Classification Between 12 and 24 Months

Among episodes with evaluable PROMs at both 12 m and 24 m, a stability class was derived to characterize longitudinal trajectories. The stability framework distinguished stable-high, late decline, late improvement, persistent low, and mixed/indeterminate patterns. Stability classification was anchored in the direction of change between 12 m and 24 m using the same MID thresholds and in changes in the traffic-light category.

### 2.6. Covariates and Clinical Context

Demographic and clinical covariates were extracted from the structured dataset and summarized by institution. Variables included age, sex, tumor site group, histology group, grade, margin status, treatment exposures (radiotherapy and chemotherapy), and a surgical complexity proxy where available. Because PROM outcomes in sarcoma may be influenced by disease course and treatment intensity, institution-level comparisons were interpreted as feasibility and benchmarking signals rather than causal treatment effects.

### 2.7. Statistical Analysis

Descriptive statistics were used to summarize cohort characteristics, evaluability at baseline and locked follow-up windows, EQ-5D-5L index and EQ-VAS distributions, MID-based change categories, traffic-light outputs, and stability classes. Continuous variables are reported as median [IQR] and categorical variables as *n* (%), unless otherwise stated.

Institution-level comparisons (Institution A vs. Institution B) were performed using non-parametric tests for continuous variables and contingency-table tests for categorical variables, with two-sided *p*-values reported. Given the feasibility and benchmarking focus of the study, analyses emphasized evaluability, distributional patterns, and interpretable effect direction. Exploratory multivariable regression modeling to assess predictors of PROM RED status was prespecified; however, given limited event counts in the evaluable subsets and the resulting vulnerability to model instability, overfitting, and sensitivity to influential observations, these analyses are not reported in the present manuscript and are reserved for subsequent work. Any future use of such models in this setting should be understood as exploratory and hypothesis-generating rather than causal or definitive.

## 3. Results

### 3.1. Cohort Composition, Hierarchical T0, and PROM Evaluability

The final analytic cohort comprised 729 malignant and intermediate episodes with a valid hierarchical T0, including 410 episodes from Institution A and 319 episodes from Institution B, based on the prespecified histology behavior mapping shown in Table S1; the corresponding tier-audit summary is provided in Table S2. Across the combined cohort, T0 was surgery-based in the majority of episodes (663/729, 91.0%), followed by radiotherapy-based anchoring in 49/729 (6.7%) and systemic-treatment anchoring in 17/729 (2.3%). Institution B had a lower proportion of surgery-based T0 and a higher proportion of radiotherapy- or systemic-based T0 than Institution A (*p* = 0.014) (Table 1). Because the hierarchical anchor reflects the recorded start of definitive treatment in heterogeneous real-world care pathways, this between-institution difference in T0 composition may also reflect differences in case-mix, treatment sequencing, referral structure, or disease complexity rather than a simple institutional effect; it may also contribute to variation in observed PRO distributions.

PROM evaluability in the prespecified locked windows was feasible but incomplete. Baseline PROMs were available for 107/729 episodes (14.7%), including 64/410 (15.6%) in Institution A and 43/319 (13.5%) in Institution B (*p* = 0.420). At 12 months, evaluable PROMs were available for 119/729 episodes (16.3%), including 75/410 (18.3%) in Institution A and 44/319 (13.8%) in Institution B (*p* = 0.103). At 24 months, evaluable PROMs were available for 84/729 episodes (11.5%), including 56/410 (13.7%) in Institution A and 28/319 (8.8%) in Institution B (*p* = 0.041) (Table 1). Baseline assessments were predominantly performed pre-treatment in both institutions, although Institution A showed a more negative median baseline offset from T0 than Institution B (−42 [−61, −21] vs. −23 [−51, −9] days; *p* = 0.017), whereas Institution B more frequently required fallback early-post baseline assignment (9.4% vs. 5.4%; *p* = 0.036) (Table 1). Because evaluability was limited at all timepoints, subsequent traffic light distributions should be interpreted as reflecting the evaluable subset within the prespecified windows rather than the full eligible cohort.

### 3.2. VOS PROM Traffic-Light Classification at 12 and 24 Months

**Figure 1:**
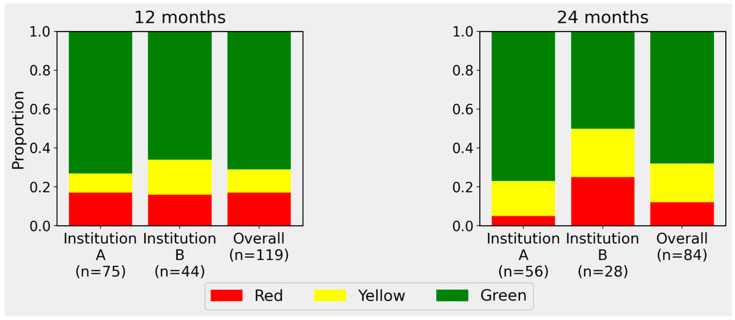
VOS PROM traffic-light distributions at locked 12- and 24-month follow-up windows by institution and overall cohort. Stacked bars show the proportion of episodes classified as GREEN, YELLOW, or RED at 12 months (**Left Panel**) and 24 months (**Right Panel**). Episodes were considered evaluable if at least one EQ-5D-5L index and/or EQ-VAS value was available within the respective locked window (12 months: 180–365 days; 24 months: 660–820 days), both anchored to hierarchical T0. Thresholds were defined as follows: RED if EQ-5D-5L index < 0.75 or EQ-VAS < 50; GREEN if EQ-5D-5L index ≥ 0.85 and EQ-VAS ≥ 70; otherwise YELLOW. *n* indicates the number of evaluable episodes in each group.

Applying the prespecified VOS PROM traffic-light classification, the majority of evaluable episodes were assigned to the GREEN category at both follow-up timepoints; however, the institutional separation was more pronounced at 24 months than at 12 months (Figure 1; Table 2).

At 12 months, Institution A showed 55/75 GREEN episodes (73.3%), 7/75 YELLOW episodes (9.3%), and 13/75 RED episodes (17.3%). Institution B showed 29/44 GREEN episodes (65.9%), 8/44 YELLOW episodes (18.2%), and 7/44 RED episodes (15.9%). The overall 12-month distribution was 84/119 GREEN (70.6%), 15/119 YELLOW (12.6%), and 20/119 RED (16.8%), with no statistically significant difference between institutions (*p* = 0.373) (Table 2).

At 24 months, Institution A showed 43/56 GREEN episodes (76.8%), 10/56 YELLOW episodes (17.9%), and 3/56 RED episodes (5.4%), whereas Institution B showed 14/28 GREEN episodes (50.0%), 7/28 YELLOW episodes (25.0%), and 7/28 RED episodes (25.0%). The overall 24-month distribution was 57/84 GREEN (67.9%), 17/84 YELLOW (20.2%), and 10/84 RED (11.9%). In contrast to the 12-month analysis, the institutional difference at 24 months was statistically significant (*p* = 0.014) (Table 2; Figure 1). Thus, the categorical traffic-light framework identified a clearer cross-institution difference in traffic-light distributions at 24 months than at 12 months, despite only modest differences in absolute medians; however, this pattern should be interpreted cautiously in light of between-institution differences in anchor composition and case-mix.

### 3.3. EQ-5D-5L Status at Baseline and Locked Follow-Up Windows

Baseline EQ-5D-5L index values were high overall, but differed between institutions. Institution A showed a higher baseline median EQ-5D-5L index than Institution B (1.00 [0.90, 1.00] vs. 0.85 [0.75, 0.95]; *p* = 0.002), whereas baseline EQ-VAS did not differ significantly (80 [50, 90] vs. 70 [50, 90]; *p* = 0.444) (Table 2).

**Figure 2:**
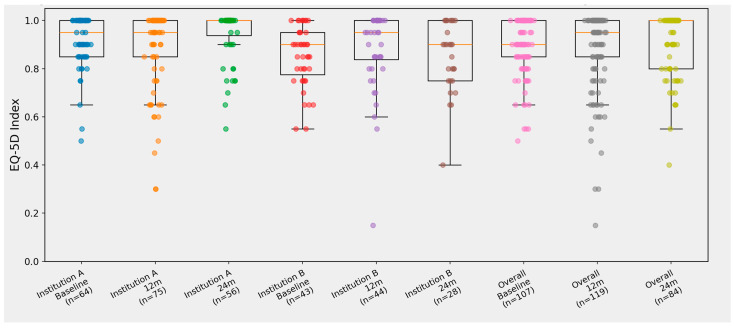
EQ-5D-5L index distributions at baseline, 12 months, and 24 months in the malignant and intermediate cohort. Boxplots with overlaid individual data points show the distribution of EQ-5D-5L index values at baseline, 12 months, and 24 months for Institution A, Institution B, and the overall cohort. Timepoints were assigned using prespecified locked windows anchored to hierarchical T0 (baseline: −90 to +14 days preferred, with +15 to +90 days fallback; 12 months: 180–365 days; 24 months: 660–820 days). n indicates the number of episodes with an available EQ-5D-5L index value in the respective time window. Horizontal reference lines indicate the prespecified EQ-5D-5L index thresholds used for traffic-light classification (0.75 and 0.85).

At 12 months, EQ-5D-5L index distributions remained concentrated in the upper range in both institutions, with median values of 1.00 [0.85, 1.00] in Institution A and 0.95 [0.80, 1.00] in Institution B (*p* = 0.624). EQ-VAS values at 12 months were similarly high, with medians of 90 [79, 95] in Institution A and 80 [65, 93.5] in Institution B (*p* = 0.731) (Table 2). At 24 months, the median EQ-5D-5L index remained high in both institutions (1.00 [0.95, 1.00] in Institution A; 0.90 [0.75, 1.00] in Institution B; *p* = 0.189), as did EQ-VAS (85.5 [79.5, 91.0] vs. 77.0 [50.0, 91.5]; *p* = 0.973) (Table 2). However, as shown in Figure 2, Institution B displayed broader dispersion and a more pronounced lower tail at both follow-up windows, with several individual episodes remaining well below the high-function range despite only modest differences in summary statistics. The combined cohort showed intermediate distributions, with the lower tail largely driven by Institution B (Figure 2).

### 3.4. Change from Baseline and MID-Based Improvement/Deterioration

Among episodes with baseline and follow-up PROMs available, median change scores were generally modest and did not differ significantly between institutions. At 12 months, median change in EQ-5D-5L index was 0.00 [−0.075, 0.125] in Institution A and 0.00 [−0.025, 0.150] in Institution B (*p* = 0.812), while the median change in EQ-VAS was 5.5 [−5.5, 14.5] and 10.0 [−6.5, 35.5], respectively (*p* = 0.548) (Table 2). MID-based improvement at 12 months was observed in 17/28 episodes (60.7%) in Institution A and 16/25 episodes (64.0%) in Institution B, whereas MID-based deterioration occurred in 15/28 (53.6%) and 11/25 (44.0%), respectively; these proportions did not differ significantly between institutions (Table 2).

At 24 months, the median EQ-5D-5L index change was 0.10 [0.00, 0.15] in Institution A and −0.025 [−0.125, 0.15] in Institution B (*p* = 0.294). Median EQ-VAS change was 11.5 [3.0, 42.0] in Institution A and 9.5 [−1.5, 20.5] in Institution B (*p* = 0.612) (Table 2). MID-based improvement at 24 months was observed in 16/18 episodes (88.9%) in Institution A and 8/12 episodes (66.7%) in Institution B, while deterioration occurred in 5/18 (27.8%) and 6/12 (50.0%), respectively. Again, these differences did not reach statistical significance (Table 2). Overall, MID-based change categories were informative for contextualizing patient trajectories, but they discriminated institutions less strongly than the traffic-light outputs at 24 months.

### 3.5. Stability Classification Between 12 and 24 Months

Among episodes evaluable at both 12 and 24 months, stability classification was available for 46 episodes (30 in Institution A and 16 in Institution B). Stable high trajectories were observed in 6/30 episodes (20.0%) in Institution A and 1/16 episodes (6.3%) in Institution B. Late decline occurred in 7/30 episodes (23.3%) in Institution A and 6/16 episodes (37.5%) in Institution B, whereas late improvement occurred in 13/30 (43.3%) and 4/16 (25.0%), respectively. Persistent low trajectories were rare overall and occurred only in Institution B (2/16, 12.5%). Mixed trajectories were observed in 4/30 (13.3%) in Institution A and 3/16 (18.8%) in Institution B. Although the overall distribution of stability classes did not reach statistical significance (*p* = 0.139), the pattern suggested more persistent adverse or declining trajectories in Institution B and more late improvement in Institution A (Table 2).

## 4. Discussion

This study shows that a prespecified, rule-based EQ-5D-5L framework can translate irregular real-world PROM data into an interpretable patient-perspective signal for sarcoma care benchmarking within an evaluable real-world subset [10]. By anchoring episodes to a hierarchical T0 and evaluating PROMs within locked baseline, 12-month, and 24-month windows, we were able to generate time-consistent traffic-light outputs and longitudinal stability patterns despite incomplete routine-care capture [34,48,49]. The principal findings are threefold. First, PROM evaluation in locked windows was operationally feasible, although markedly incomplete. Second, the resulting traffic-light classification identified heterogeneity in patient-perceived outcomes within the evaluable subset, with the majority of evaluable episodes remaining in the acceptable range but with a clinically relevant lower tail. Third, the institutional separation was more pronounced at 24 months than at 12 months, suggesting that longer-term follow-up may be more informative than a single early survivorship snapshot when patient-perspective burden is used as a benchmarking signal.

The stronger signal at 24 months is clinically plausible. Sarcoma pathways often involve delayed recovery trajectories, late effects of radiotherapy, repeated procedures, chronic pain syndromes, or disease-related events that may not be fully expressed at 12 months [50,51,52]. A 12-month assessment may therefore capture patients during ongoing functional adaptation rather than in a more consolidated survivorship state. In contrast, the 24-month window appears to discriminate more clearly between episodes that remain in an acceptable health-state range and those that evolve into persistent or late-emerging burden [53]. This is further supported by the stability analysis, which suggested more late-decline and persistent adverse trajectories in Institution B and more late improvement in Institution A, even though formal statistical separation of stability classes remained limited by the smaller longitudinally evaluable subset [54]. Taken together, these findings support the inclusion of a later survivorship checkpoint in sarcoma outcome monitoring and caution against relying exclusively on earlier follow-up for patient-perspective benchmarking.

A central implication of this work is that PROMs can be made operationally steerable for value-based sarcoma care when they are treated as a measurement discipline rather than as ad hoc descriptive endpoints [27]. In practical terms, steerability requires four elements that are often under-specified in PROM reporting: (i) a clinically meaningful anchoring event, here defined as hierarchical T0; (ii) prespecified evaluation windows that avoid shifting denominators and moving targets; (iii) transparent reporting of evaluability, so that missingness is explicit rather than implicit; and (iv) an interpretable output that can be acted upon in routine governance [55]. The traffic-light classification used here does not replace detailed PROM reporting [34]. Nor should the traffic-light categories be understood as fully validated patient-anchored states; rather, they represent a pragmatic operational layer intended to support interpretation and structured review in routine care. In this form, the framework condenses EQ-5D-5L index and EQ-VAS into an interpretable patient-perspective signal that supports cross-institution review, pathway benchmarking, and iterative quality improvement, while still allowing deeper inspection of absolute values and change scores where needed [56]. In this sense, the present study is not merely descriptive; it provides an initial operational module for integrating patient-reported outcomes into a value-based sarcoma care framework.

The sarcoma-specific context is important for interpreting the traffic-light categories. In many cancer settings, PROM improvement is intuitively aligned with treatment success [57]. In sarcoma, by contrast, treatment is often oncologically necessary even when it causes functional loss, and many patients begin treatment with near-normal baseline status [58]. Under these conditions, value cannot be reduced to simple improvement scores [59]. Instead, it must be understood as the ability to maintain or recover an acceptable health state despite necessary morbidity. This is the rationale for combining absolute state with meaningful change relative to baseline. Within this framework, GREEN indicates maintained or restored acceptability, RED indicates a clearly unacceptable health state, and YELLOW identifies the clinically important intermediate zone in which absolute status is not frankly poor but deterioration, mixed signals, or uncertainty warrant closer review [60]. In sarcoma care, this YELLOW zone is arguably where governance can be most useful, as it may identify episodes requiring intensified rehabilitation, symptom control, psychosocial support, or pathway-level audit before persistent adverse trajectories become entrenched. From an operational perspective, a YELLOW signal should not be interpreted as a definitive adverse outcome, but rather as a prompt for structured review. Illustrative action options include repeat PROM assessment within a defined interval, focused review of pain and function-limiting symptoms, rehabilitation or physiotherapy reassessment, screening for psychosocial distress, and multidisciplinary review when the signal persists, worsens, or is clinically discordant with the expected recovery course. These examples are intended as pragmatic implementation options rather than as validated mandatory intervention rules.

The cross-institution differences observed in this study should be interpreted as benchmarking signals rather than performance rankings [61]. The present design was not intended to establish causality, nor was it fully risk-adjusted for all clinically relevant sources of heterogeneity. Differences in case-mix, treatment complexity, late effects, oncologic course, referral patterns, or local workflow may all contribute to the observed separation, particularly at 24 months [42]. In particular, Institution B had a lower proportion of surgery-based T0 and a higher proportion of radiotherapy- or systemic-based anchoring, which may indicate differences in treatment pathway structure or underlying clinical complexity rather than differences in treatment quality per se. This is relevant because surgery-, radiotherapy-, and systemic-based anchors may capture patients at different phases of care and with different expected functional burdens, such that anchor variation may contribute to differences in observed PRO distributions. Importantly, the findings show that the methodology is capable of detecting a signal under routine-care conditions; they do not establish that one institution provides better care than the other [62,63]. In this respect, the traffic-light framework should be viewed as a trigger for structured inquiry rather than as an endpoint in itself. Its main function is to make otherwise diffuse patient-reported data visible and comparable enough to inform network-level learning, while remaining interpretable in conjunction with standard clinical and oncologic assessment. PROM-based traffic-light signals should not be interpreted in isolation, but alongside standard clinical and oncologic assessment. In sarcoma care, imaging—particularly MRI—remains essential for diagnosis, local staging, treatment monitoring, and surveillance for recurrence. Future integration of PROM signals with imaging and other objective clinical markers may further strengthen value-based interpretation, but such multimodal integration was beyond the scope of the present operationalization study.

A further interpretive constraint is that PROM missingness in this setting is unlikely to be fully random. Patients with higher symptom burden, recurrence, deterioration, treatment fatigue, or lower engagement with follow processes may plausibly be less likely to complete questionnaires over time. If so, the observed traffic-light distributions may under- or overestimate the true proportion of RED, YELLOW, and GREEN episodes, particularly at later follow-up. The present findings should therefore be understood as demonstrating the operational applicability of the framework within the evaluable subset rather than unbiased full-cohort benchmarking.

### Limitations

Several limitations should be acknowledged. Most importantly, PROM evaluability was incomplete, and the reported outcomes therefore reflect evaluable episodes within prespecified windows rather than the entire eligible cohort [40]. Systematic differences between evaluable and non-evaluable episodes cannot be excluded, and non-random missingness may have influenced the apparent benchmarking signal, particularly at later follow-up [64]. Although this is a limitation, it is also an important empirical finding: incomplete and irregular PROM capture is itself a major constraint on reliable benchmarking. Second, while the present cohort was harmonized at the level of malignant and intermediate tumor behavior, several sarcoma-domain variables that are likely to influence functional outcomes and PROM interpretation—most notably bone versus soft tissue disease, anatomical reconstruction exposure and complexity, limb-salvage versus amputation context, and more granular procedure-related morbidity—were not included in the primary analysis because they were not yet sufficiently standardized across institutions in the current data export [65]. Third, the traffic-light thresholds and MID cutoffs were prespecified as pragmatic operational rules informed by clinical reasoning and external EQ-5D-5L reference concepts, but they are not yet validated against sarcoma-specific patient-anchored value measures such as treatment worthwhileness, recovery burden, or willingness to repeat [66]. Finally, although exploratory regression modeling was prespecified, the evaluable event counts in some subsets remain limited, and any such models would be vulnerable to instability, overfitting, and distortion by influential observations. The present manuscript therefore focuses primarily on feasibility and signal detection rather than inferential modeling [67]. Accordingly, the present study does not circumvent the broader methodological limitations of exploratory multivariable regression highlighted in recent methodological literature, and no causal or definitive predictor interpretation is claimed [68,69]. In addition, generalizability remains limited to comparable real-world sarcoma care settings with similar PROM implementation maturity and data structure.

These limitations also define the immediate next steps. First, PROM capture should be strengthened as a first-order implementation target, with operational standards aligned to T0 and the locked evaluation windows used here [39]. During the study period, PROM capture reflected partial real-world implementation and was not yet uniformly embedded across all involved treatment disciplines. Improving capture will require direct integration into routine outpatient workflows, predefined collection windows, clearer assignment of team responsibility, digital reminders or repeat prompts, and greater visibility of PROM results for treating teams so that questionnaire completion is linked to clinically actionable use rather than perceived as a parallel documentation task. Where follow-up questionnaires are missed, repeat capture within the prespecified window or at the next clinically relevant contact should be considered as part of a pragmatic recovery strategy. Second, subsequent analyses should move toward risk-adjusted benchmarking by incorporating clinically relevant sarcoma-domain variables such as bone versus soft tissue disease, anatomical site, reconstruction and re-reconstruction complexity, limb salvage versus amputation context, site-specific morbidity, recurrence context, multimodality treatment intensity, and more granular oncologic status [70]. Once harmonized across sites, these variables should serve as the structural backbone of the next risk-adjustment layer, enabling fairer interpretation of traffic light distributions in relation to expected functional burden. Only after improved variable harmonization and larger evaluable subsets should exploratory multivariable modeling be revisited for hypothesis-generating purposes. Even in that setting, such models would require cautious interpretation and should not be used to infer causal or stable risk factors without stronger design and prespecification. Third, the framework should be stress-tested by sensitivity analyses around thresholds and window definitions and then prospectively pilot-validated against direct patient-anchored outcomes, including perceived treatment worthwhileness, recovery burned, supportive care needs, and qualitative feedback on whether the assigned traffic-light category matches the patient’s lived experience [71]. Such steps would allow the present module to evolve from a feasibility-and-signal tool into a more robust governance instrument within a learning health system [72].

## 5. Conclusions

In conclusion, this study shows that a rule-based EQ-5D-5L framework, anchored to hierarchical T0 and evaluated in locked windows, can produce an interpretable patient-perspective signal for benchmarking in real-world sarcoma care. The approach was operationally feasible within the evaluable subset, revealed clinically meaningful heterogeneity, and identified a clearer cross-institution separation at 24 months than at 12 months. At the same time, incomplete PROM capture remains a major implementation bottleneck and underscores the need for workflow-integrated PROM collection strategies. Taken together, these findings support the use of structured PROM modules not only for descriptive outcome reporting, but as an operational foundation for value-based sarcoma care and future network-level learning.

## Figures and Tables

**Table 1:** Cohort characteristics, hierarchical time anchoring (T0), and PROM evaluability by institution.

Variable	Institution A	Institution B	Overall	*p* (A vs. B)
Cohort and hierarchical T0				
Episodes with hierarchical T0 (malignant + intermediate)	410	319	729	
T0 source: Surgery, *n* (%)	384 (93.66%)	279 (87.46%)	663 (90.95%)	0.014
T0 source: Radiotherapy, *n* (%)	20 (4.88%)	29 (9.09%)	49 (6.72%)	
T0 source: Systemic, *n* (%)	6 (1.46%)	11 (3.45%)	17 (2.33%)	
Behavior tier: Malignant, *n* (%)	287 (70.00%)	211 (66.14%)	498 (68.31%)	0.303
Behavior tier: Intermediate, *n* (%)	123 (30.00%)	108 (33.86%)	231 (31.69%)	
PROM evaluability and timing				
Baseline PROM available	64 (15.61%)	43 (13.48%)	107 (14.68%)	0.420
12 m PROM available	75 (18.29%)	44 (13.79%)	119 (16.32%)	0.103
24 m PROM available	56 (13.66%)	28 (8.78%)	84 (11.52%)	0.041
Baseline offset from T0 (days), median [IQR]	−42 [−61, −21]	−23 [−51, −9]	−37 [−58, −9]	0.017
Early-post baseline (>+14 d–90 d), *n* (%)	22 (5.37%)	30 (9.40%)	52 (7.13%)	0.036
12 m offset from T0 (days), median [IQR]	289 [245, 336]	298 [259, 341]	301 [278, 341]	0.125
24 m offset from T0 (days), median [IQR]	732.5 [700, 768]	729.5 [698.5, 766]	736 [700, 768]	0.951
Demographic characteristics				
Sex: Female, *n* (%)	190 (46.34%)	153 (47.96%)	343 (47.05%)	0.719
Sex: Male, *n* (%)	220 (53.66%)	166 (52.04%)	386 (52.95%)	
Sex: Unknown, *n* (%)	0 (0.00%)	0 (0.00%)	0 (0.00%)	
Tumor status and treatment context				
Margin status: R0, *n* (%)	238 (61.98%)	154 (48.28%)	392 (53.77%)	<0.001
Margin status: R1, *n* (%)	68 (17.71%)	58 (18.18%)	126 (17.28%)	
Margin status: R2, *n* (%)	14 (3.65%)	12 (3.76%)	26 (3.57%)	
Margin status: Unknown, *n* (%)	64 (16.67%)	95 (29.78%)	159 (21.81%)	
Metastasis at diagnosis: Yes, *n* (%)	48 (11.71%)	36 (11.29%)	84 (11.52%)	0.453
Metastasis at diagnosis: No, *n* (%)	351 (85.61%)	269 (84.33%)	620 (85.05%)	
Metastasis at diagnosis: Unknown, *n* (%)	11 (2.68%)	14 (4.39%)	25 (3.43%)	
Metastasis during follow-up: Yes, *n* (%)	146 (35.61%)	105 (32.92%)	251 (34.43%)	0.301
Metastasis during follow-up: No, *n* (%)	230 (56.10%)	177 (55.49%)	407 (55.83%)	
Metastasis during follow-up: Unknown, *n* (%)	34 (8.29%)	37 (11.60%)	71 (9.74%)	
Any radiotherapy recorded: Yes, *n* (%)	219 (53.41%)	155 (48.59%)	374 (51.30%)	0.428
Any radiotherapy recorded: No, *n* (%)	155 (37.80%)	132 (41.38%)	287 (39.37%)	
Any radiotherapy recorded: Unknown, *n* (%)	36 (8.78%)	32 (10.03%)	68 (9.33%)	
Any chemotherapy recorded: Yes, *n* (%)	118 (28.78%)	97 (30.41%)	215 (29.49%)	0.492
Any chemotherapy recorded: No, *n* (%)	251 (61.22%)	183 (57.37%)	434 (59.53%)	
Any chemotherapy recorded: Unknown, *n* (%)	41 (10.00%)	39 (12.23%)	80 (10.97%)	
Whoops surgery: Yes, *n* (%)	83 (20.24%)	74 (23.20%)	157 (21.54%)	0.023
Whoops surgery: No, *n* (%)	298 (72.68%)	206 (64.58%)	504 (69.14%)	
Whoops surgery: Unknown, *n* (%)	29 (7.07%)	39 (12.23%)	68 (9.33%)	

Episodes were included if classified as malignant or intermediate by the prespecified three-tier histology mapping (Table S1) and if a valid hierarchical time anchor (T0) was available. T0 was defined hierarchically as the start of definitive treatment: index surgery date when available; otherwise radiotherapy start date; otherwise systemic anticancer therapy start date where a valid and interpretable start date was available. Baseline and follow-up PROM evaluability were defined as the availability of at least one EQ-5D-5L index value and/or EQ-VAS score within the prespecified windows (baseline: −90 to +14 days preferred, with +15 to +90 days fallback; 12 months: 180–365 days; 24 months: 660–820 days). Continuous variables are reported as median [IQR] and categorical variables as *n* (%). *p*-values compare Institution A versus Institution B.

**Table 2:** EQ-5D status, MID-based change, and VOS PROM traffic-light outputs at locked 12- and 24-month windows.

Outcome	Institution A	Institution B	Overall	*p* (A vs. B)
Absolute PROM status				
Baseline: EQ-5D index, median [IQR] (*n*)	1.00 [0.90, 1.00] (*n* = 64)	0.85 [0.75, 0.95] (*n* = 43)	0.95 [0.85, 1.00] (*n* = 107)	0.002
Baseline: EQ-5D VAS, median [IQR] (*n*)	80 [50, 90] (*n* = 64)	70 [50, 90] (*n* = 43)	80 [50, 90] (*n* = 107)	0.444
12 months: EQ-5D index, median [IQR] (*n*)	1.00 [0.85, 1.00] (*n* = 75)	0.95 [0.80, 1.00] (*n* = 44)	1.00 [0.85, 1.00] (*n* = 119)	0.624
12 months: EQ-5D VAS, median [IQR] (*n*)	90 [79, 95] (*n* = 75)	80 [65, 93.5] (*n* = 44)	90 [79, 95] (*n* = 119)	0.731
24 months: EQ-5D index, median [IQR] (*n*)	1.00 [0.95, 1.00] (*n* = 56)	0.90 [0.75, 1.00] (*n* = 28)	1.00 [0.80, 1.00] (*n* = 84)	0.189
24 months: EQ-5D VAS, median [IQR] (*n*)	85.5 [79.5, 91.0] (*n* = 56)	77.0 [50.0, 91.5] (*n* = 28)	85.0 [71.5, 96.0] (*n* = 84)	0.973
MID-based change from baseline				
Change to 12 m: ΔEQ-5D index, median [IQR] (*n*)	0.00 [−0.075, 0.125] (*n* = 28)	0.00 [−0.025, 0.150] (*n* = 25)	0.00 [−0.075, 0.100] (*n* = 53)	0.812
Change to 12 m: ΔEQ-VAS, median [IQR] (*n*)	5.5 [−5.5, 14.5] (*n* = 28)	10.0 [−6.5, 35.5] (*n* = 25)	10.0 [−5.5, 29.5] (*n* = 53)	0.548
Change to 12 m: clinically meaningful improvement (≥MID)	17 (60.71%)	16 (64.00%)	33 (62.26%)	1.000
Change to 12 m: clinically meaningful deterioration (≤−MID)	15 (53.57%)	11 (44.00%)	26 (49.06%)	0.586
Change to 24 m: ΔEQ-5D index, median [IQR] (*n*)	0.10 [0.00, 0.15] (*n* = 18)	−0.025 [−0.125, 0.15] (*n* = 12)	0.075 [−0.05, 0.15] (*n* = 30)	0.294
Change to 24 m: ΔEQ-VAS, median [IQR] (*n*)	11.5 [3.0, 42.0] (*n* = 18)	9.5 [−1.5, 20.5] (*n* = 12)	9.5 [0.0, 42.0] (*n* = 30)	0.612
Change to 24 m: clinically meaningful improvement (≥MID)	16 (88.89%)	8 (66.67%)	24 (80.00%)	0.184
Change to 24 m: clinically meaningful deterioration (≤−MID)	5 (27.78%)	6 (50.00%)	11 (36.67%)	0.266
PROM traffic-light outputs				
PROM traffic light at 12 m: GREEN	55 (73.33%)	29 (65.91%)	84 (70.59%)	0.373
PROM traffic light at 12 m: YELLOW	7 (9.33%)	8 (18.18%)	15 (12.61%)	
PROM traffic light at 12 m: RED	13 (17.33%)	7 (15.91%)	20 (16.81%)	
PROM traffic light at 12 m: evaluable *n*	75	44	119	
PROM traffic light at 24 m: GREEN	43 (76.79%)	14 (50.00%)	57 (67.86%)	0.014
PROM traffic light at 24 m: YELLOW	10 (17.86%)	7 (25.00%)	17 (20.24%)	
PROM traffic light at 24 m: RED	3 (5.36%)	7 (25.00%)	10 (11.90%)	
PROM traffic light at 24 m: evaluable *n*	56	28	84	
24-month stability classification				
24 m stability class (among those with both 12 m and 24 m): STABLE_HIGH	6 (20.00%)	1 (6.25%)	7 (15.22%)	0.139
24 m stability class: LATE_DECLINE	7 (23.33%)	6 (37.50%)	13 (28.26%)	
24 m stability class: LATE_IMPROVEMENT	13 (43.33%)	4 (25.00%)	17 (36.96%)	
24 m stability class: PERSISTENT_LOW	0 (0.00%)	2 (12.50%)	2 (4.35%)	
24 m stability class: MIXED	4 (13.33%)	3 (18.75%)	7 (15.22%)	
24 m stability class: evaluable *n*	30	16	46	

EQ-5D-5L index and EQ-VAS are summarized at baseline, 12 months, and 24 months using prespecified locked windows anchored to hierarchical T0 (baseline: −90 to +14 days preferred, with +15 to +90 days fallback; 12 months: 180–365 days, target 270 days; 24 months: 660–820 days, target 730 days). Clinically meaningful change was defined using prespecified MID thresholds (ΔEQ-5D-5L index ≥ +0.03 or ≤−0.03; ΔEQ-VAS ≥ +5 or ≤−5). PROM traffic-light outputs were derived using the rule-based VOS framework: RED if EQ-5D-5L index < 0.75 or EQ-VAS < 50; GREEN if EQ-5D-5L index ≥ 0.85 and EQ-VAS ≥ 70; otherwise YELLOW. Evaluable *n* indicates episodes with at least one valid EQ-5D-5L index and/or EQ-VAS value in the respective time window.

## Data Availability

The data presented in this study are available on request from the corresponding author.

## Supplementary Materials

The following supporting information can be downloaded at: https://www.mdpi.com/article/10.3390/diseases14050159/s1, Table S1: Histology three-tier behavior mapping (Benign/Intermediate/Malignant) and adjudication log; Table S2: Tier-audit summary of the final analytic cohort by institution.

## Abbreviations

The following abbreviations are used in this manuscript:

EQ-5D-5L	EuroQoL 5-Dimension
EQ-VAS	EuroQoL Visual Analogue Scale
IQR	Interquartile Range
MID	Minimal Important Difference
PROMs	Patient-Reported Outcome Measures
PRO	Patient-Reported Outcome
T0	Time Zero
VAS	Visual Analogue Scale
VOS	Value Operating System
