# Supplementary material for: Locked-Window EQ-5D-5L (Index and VAS) Benchmarking in Sarcoma Care: Rule-Based Traffic-Light Classification Across Two Institutions
Source: Diseases. 2026 Apr 30;14(5):159. doi: 10.3390/diseases14050159 (PMC13205251; doi:10.3390/diseases14050159)
Supplement: Supplementary file 1 [file diseases-14-00159-s001.zip › diseases-4252416-supplementary.pdf]

Supplementary Materials: The following supporting information can be downloaded at: [link will be generated by the journal], **Table S1: Histology three-tier behavior mapping (Benign/Intermediate/Malignant) and adjudication log; Table S2: Tier-audit summary of the final analytic cohort by institution.**

Table S1. Histology three-tier behavior mapping (Benign/Intermediate/Malignant) and adjudication log.

Histological diagnosis (raw label)	Behavior tier	Confidence	Rationale
**Malignant peripheral nerve sheath tumor**	Malignant	High	Explicit malignant sarcoma entity
**Synovial sarcoma**	Malignant	High	Explicit malignant sarcoma entity
**Epithelioid sarcoma**	Malignant	High	Explicit malignant sarcoma entity
**Clear cell sarcoma of soft tissue**	Malignant	High	Explicit malignant sarcoma entity
**Extraskeletal myxoid chondrosarcoma**	Malignant	High	Explicit malignant sarcoma entity
**Angiosarcoma**	Malignant	High	Explicit malignant sarcoma entity
**Leiomyosarcoma (bone or soft tissue)**	Malignant	High	Explicit malignant sarcoma entity
**Osteosarcoma (all subtypes)**	Malignant	High	Explicit malignant sarcoma entity
**Chondrosarcoma (all subtypes)**	Malignant	High	Explicit malignant sarcoma entity
**Ewing sarcoma**	Malignant	High	Explicit malignant sarcoma entity
**Undifferentiated pleomorphic sarcoma**	Malignant	High	Explicit malignant sarcoma entity
**Myxofibrosarcoma**	Malignant	High	Explicit malignant sarcoma entity
**Rhabdomyosarcoma**	Malignant	High	Explicit malignant sarcoma entity
**Chordoma**	Malignant	High	Malignant bone tumor
**Sarcomatoid carcinoma**	Malignant	High	Explicit malignant entity
**Atypical lipomatous tumor / well-differentiated liposarcoma**	Malignant	High	Malignant liposarcoma entity
**Dedifferentiated liposarcoma**	Malignant	High	Malignant liposarcoma entity
**Myxoid liposarcoma**	Malignant	High	Malignant liposarcoma entity
**Pleomorphic liposarcoma**	Malignant	High	Malignant liposarcoma entity
**Low-grade myofibroblastic sarcoma**	Malignant	High	Explicit malignant sarcoma entity
**Solitary fibrous tumor, malignant**	Malignant	High	Label explicitly states malignant
**Desmoid-type fibromatosis**	Intermediate	High	Locally aggressive, non-metastasizing

**Giant cell tumor of bone**	Intermediate	High	Locally aggressive, rare metastasis
**Tenosynovial giant cell tumor**	Intermediate	High	Locally aggressive entity
**Solitary fibrous tumor**	Intermediate	High	Intermediate behavior
**Desmoplastic fibroma of bone**	Intermediate	Medium	Locally aggressive, borderline
**Atypical spindle cell / pleomorphic lipomatous tumor**	Intermediate	Medium	Borderline/intermediate behavior
**Hemosiderotic fibrolipomatous tumor**	Intermediate	Medium	Intermediate behavior
**Langerhans cell histiocytosis**	Intermediate	Medium	Variable, not classic malignant sarcoma
**Mixed tumor NOS**	Unclear	Low	Insufficient specificity
**Phosphaturic mesenchymal tumor**	Unclear	Low	Behavior cannot be inferred from label alone
**Encoded multiple-diagnosis strings**	Unclear	Low	Requires episode-level clarification
**Rare unclassifiable entities**	Unclear	Low	Requires explicit pathology behavior code

Table S1. Histology three-tier behavior mapping (Benign/Intermediate/Malignant) and adjudication log.
Unique histological diagnosis lables extracted from the source datasets were harmonized and mapped to a three-tier tumor-behavior framework (Benign, Intermediate, Malignant) unsing a prespecified rule set. For each label, the assigned tier, confidence level, and rationale are provided. Lables that could not be categorized unambiguously from the recorded diagnosis string were flagged as "Unclear" and excluded from the primary malignant+intermediate cohort. The complete list of "Unclear" lables provided to enable transparent adjudication once definitive behavior coding (e.g., ICD-= behavior code or explicit pathology statement) becomes available.

Table S2. Tier-audit summary of the final analytic cohort by institution.

**Behavior tier**	**Institution A**	**Institution B**	**Overall**
**Benign, n (%)**	0 (0.00%)	0 (0.00%)	0 (0.00%)
**Intermediate, n (%)**	123 (30%)	108 (33.9%)	231 (31.7%)
**Malignant, n (%)**	287 (70%)	211 (66.1%)	498 (68.3%)
**Unclear, n (%)**	0 (0.00%)	0 (0.00%)	0 (0.00%)
**Total, n (%)**	410 (100%)	319 (100%)	729 (100%)

Table S2. Tier-audit summary of the final analytic cohort by institution.
Distribution of behavior categories after application of the prespecified histology mapping and exclusion rules. Only malignant and intermediate episodes were retained in the final analytic cohort; benign and unclear episodes were excluded prior to analysis.
